# The Impact of Variable Degrees of Freedom and Scale Parameters in Bayesian Methods for Genomic Prediction in Chinese Simmental Beef Cattle

**DOI:** 10.1371/journal.pone.0154118

**Published:** 2016-05-03

**Authors:** Bo Zhu, Miao Zhu, Jicai Jiang, Hong Niu, Yanhui Wang, Yang Wu, Lingyang Xu, Yan Chen, Lupei Zhang, Xue Gao, Huijiang Gao, Jianfeng Liu, Junya Li

**Affiliations:** 1 Laboratory of Molecular Biology and Bovine Breeding, Institute of Animal Science, Chinese Academy of Agricultural Sciences, Beijing, China; 2 Department of Animal Genetics, Breeding and Reproduction, China Agricultural University, Beijing, China; Huazhong Agricultural University, CHINA

## Abstract

Three conventional Bayesian approaches (BayesA, BayesB and BayesCπ) have been demonstrated to be powerful in predicting genomic merit for complex traits in livestock. A priori, these Bayesian models assume that the non-zero SNP effects (marginally) follow a *t*-distribution depending on two fixed hyperparameters, degrees of freedom and scale parameters. In this study, we performed genomic prediction in Chinese Simmental beef cattle and treated degrees of freedom and scale parameters as unknown with inappropriate priors. Furthermore, we compared the modified methods (BayesFA, BayesFB and BayesFCπ) with their corresponding counterparts using simulation datasets. We found that the modified methods with distribution assumed to the two hyperparameters were beneficial for improving the predictive accuracy. Our results showed that the predictive accuracies of the modified methods were slightly higher than those of their counterparts especially for traits with low heritability and a small number of QTLs. Moreover, cross-validation analysis for three traits, namely carcass weight, live weight and tenderloin weight, in 1136 Simmental beef cattle suggested that predictive accuracy of BayesFCπ noticeably outperformed BayesCπ with the highest increase (3.8%) for live weight using the cohort masking cross-validation.

## Introduction

Genomic prediction uses all available molecular markers as covariates in a linear regression model to estimate genomic breeding values for quantitative traits. This has aroused a strong interest on the explorations of genomic selection in animals and plants [1–5].

Currently, improving the accuracy of genomic breeding values has been recognized as a crucial step for genomic improvement. Various Bayesian linear regression methods have been proposed to improve the predictive accuracy in genomic selection such as BayesC [6], BayesCπ and BayesDπ [7]. To denote these Bayesian methods, Gianola *et al*. coined the term “Bayesian alphabet” using the numbers of letters of the alphabet [8]. These methods are different in the adoption of priors while sharing the same sampling model: a Gaussian distribution with a mean vector represented by a regression on numbers of markers, typically SNPs, and a residual variance $\sigma_{e}^{2}$. Bayesian Lasso used another distribution (Laplace) as the marker effect prior assumption to predict genomic estimated breeding values (GEBVs) [9–11]. Other Bayesian approaches such as BayesRS (sharing of information across populations model) [12] and BayesTA, BayesTB, and BayesTCπ (threshold models) [13] were proposed to improve the accuracy of genomic prediction.

BayesA assumes all SNPs have effects, and each SNP has its own variance, while BayesB assume that the prior distribution of SNP effects are zero with probability π, and normally distributed with a zero mean and a locus-specific variance with probability (1- π) [14]. BayesCπ treats π as an unknown parameter with a uniform (0, 1) prior distribution, and assumes all SNP effects have a common variance [7]. These methods assume the variances of SNP effects follow a scaled inverse chi-square prior distribution with degrees of freedom *v*, and a scale parameter $S_{a}^{2}$. A general consensus reveals that the full-conditional posterior distribution of a locus-specific variance adds only one to the degrees of freedom in BayesA as compared with its prior assumption, while in BayesCπ it increases the value by the number of markers that have effects in each iteration [8]. For BayesA, BayesB and BayesCπ, hyperparameter *v* is fixed (4.2 or 4), and $S_{a}^{2}$ is usually derived from an assumed additive-genetic variance [15]. However, the two fixed hyperparameters may vary in different genetic architecture [8]. Many studies have proposed some alternative priors to estimate these two hyperparameters. For instance, Habier *et al*. proposed BayesDπ by extending the BayesB method and treating *S*_*a*_ as unknown with Gamma(1,1) [7]. Yi *et al*. developed a model to estimate two hyperparameters *v* and $S_{a}^{2}$ by assigning a uniform density on 1/*v* for the range (0,1] and a uniform distribution on $S_{a}^{2}$ for the range (0,A] with A being a large number in the extent of BayesA [16]. Yang *et al*. applied a Gamma prior distribution on $S_{a}^{2}$ with parameter *α*_*s*_ = *β*_*s*_ = 0.1, and specified *v*~p (*v*) ∝(*v*+1)^-2^ for both BayesA and BayesB [17], and they further used three alternative Markov Chain Monte Carlo approaches based on Metropolis-Hastings to estimate these two hyperparameters [18]. To investigate the impact of variable degrees of freedom and scale parameters in Bayesian methods for genomic prediction in Chinese Simmental beef cattle, we treated *v* and $S_{a}^{2}$ as unknown and gave inappropriate priors in our study. These modified methods were termed as BayesFA, BayesFB and BayesFCπ, respectively.

The objectives of this study were to: (1) investigate the impact of variable *v* and $S_{a}^{2}$ on the predictive accuracy for live weight, carcass weight and tenderloin weight in Chinese Simmental beef cattle using three modified methods (BayesFA, BayesFB and BayesFCπ); (2) explore relationships between these two hyperparameters and genetic architecture especially for the number of QTL in simulation study; (3) compare the predictive accuracies among these methods (GBLUP, BayesA, BayesB, BayesCπ, BayesFA, BayesFB and BayesFCπ) using cross-validation scheme in Chinese Simmental beef cattle population.

## Materials and Methods

### Ethics statement

All animals were treated following the guidelines for the experimental animals established by the Council of China. Animal experiments were approved by the Science Research Department of the Institute of Animal Science, Chinese Academy of Agricultural Sciences (CAAS) (Beijing, China).

### Statistical Model

The general statistical model is as follow:

y=Xb+Zα+e (1)

Here, **y** is an *N*×1 (*N* = number of observations) vector of phenotypes, **X** is an incidence matrix of fixed effects in **b**, **Z** is an *N*×*P* (*P* = number of SNPs) matrix of marker codes (genotypes were coded as 0, 1 or 2). **α** is an *P*×1 vector of SNP effects. **e** is a vector of residuals. It is assumed that **y** is conditionally independent and distributed as

$$\left(y|b,\alpha ,\sigma_{e}^{2})∼N(Xb+Z\alpha ,I\sigma_{e}^{2}\right)$$ (2)

### Prior specifications

The prior assumption for *α*_*j*_ depends on the variance $\sigma_{j}^{2}$ and the probability π. In BayesA and BayesFA, all SNPs have effects and their prior distributions were normal distribution with mean 0 and variance $\sigma_{j}^{2}$, and each SNP has a specific variance, *i.e.,* $\sigma_{j}^{2}∼x^{-2}(v,vS_{a}^{2})$. $\sigma_{j}^{2}$ denotes that SNP *j* has its own variance, and each of these variances has a scaled inverse chi-square distribution with the parameters of *v* and $S_{a}^{2}$. In BayesB and BayesFB, two-component mixture with one component being $t(0,v,S_{a}^{2})$ and the other component being a spike at 0 are provided, *i*.*e*.,

$$\begin{array}{l} \alpha_{j}|\pi ,\sigma_{j}^{2}∼(idd)\{\begin{array}{c} 0\begin{array}{cc} & \;wtih_{}\;probability_{}\;\pi \end{array}\\ {{}^{}}_{}\;N(0,\sigma_{j}^{2})\begin{array}{cc} & \;with_{}\;probability^{}\;(1-\pi ) \end{array} \end{array}\\ where_{}j=1,\ldots ..,q\\ \sigma_{j}^{2}|v,S_{a}^{2}∼(idd)vS_{a}^{2}x_{v}^{-2}\;when_{}\;\sigma_{j}^{2}>0 \end{array}$$ (3)

Here, π represents the proportion of SNPs with no associated genetic effects on the trait of interest. BayesCπ and BayesFCπ also assume only a small proportion of SNPs have effects with probability (1-π) and the variances of SNP effects $\sigma_{j}^{2}=\sigma_{a}^{2}$, where $\sigma_{a}^{2}$ is the common variance of all the SNP effects. In BayesA, BayesB and BayesCπ, $S_{a}^{2}$ is normally derived from the Eq (4),

$$S_{a}^{2}=\frac{\left(v-2)\;*\;E(\sigma_{j}^{2}\right)}{v}$$ (4)

where *v* is fixed value (4.2) [14].

In present study, two hyperparameters (*v* and $S_{a}^{2}$) was further treated as unknown with inappropriate priors. *v* was treated with gamma (4, 1) distribution in BayesFB, and exponential (0.25) distribution in BayesFA and BayesFCπ. $S_{a}^{2}$ was treated as unknown with the prior gamma (1, 1) distribution in these modified methods, which was under same assumption as in BayesDπ [7]. BayesFCπ treated π as unknown with uniform (0, 1).

### Inference of model hyperparameters

Markov Chain Monte Carlo algorithms were used in the modified methods (BayesFA, BayesFB and BayesFCπ) to sample the parameters. The posterior distribution of SNP effects implemented in these modified methods were the same as those of BayesA, BayesB [14] and BayesCπ[7]. The posterior distributions of the variances of SNP effects in BayesA and BayesFA were scaled inverse chi-square distribution $\left[\sigma_{j}^{2}|v+1,(vS_{a}^{2}+\alpha_{j}^{2})/(v+1)\right]$. BayesFB used Metropolis Hastings algorithm to sample SNP effects and variances as in BayesB. And the posterior distribution of the variances of SNP effects in BayesCπ and BayesFCπ were inverse chi-square with degrees of freedom *v* + *q* and scale $\left(vS_{a}^{2}+\sum_{j=1}^{q}\alpha_{j}^{2})/(v+q\right)$, where *q* was the number of SNPs fitted with non-zero effect in current iteration.

Full conditional posterior distribution of *v* in BayesFA and BayesFCπ (5a), and BayesFB (5b) were articulated as follows:

$$\begin{array}{l} f(v|ELSE)\propto f(\sigma_{j}^{2}|S_{a}^{2},v)f(v)\\ =\prod_{j=1}^{q}\frac{{\left(vS_{a}^{2}/2\right)}^{v/2}\exp(-\frac{vS_{a}^{2}}{2\sigma_{j}^{2}})}{\Gamma (v/2)\;*\;{\left(\sigma_{j}^{2}\right)}^{1+v/2}}\lambda \;*\;\exp(-\lambda v)\\ \propto {\left(v/2\right)}^{qv/2}{\left[\Gamma (v/2)\right]}^{-q}\exp(-\eta v)\\ \eta =\frac{1}{2}\sum_{j=1}^{q}\ln(\frac{\sigma_{j}^{2}}{S_{a}^{2}})+\frac{1}{2}\sum_{j=1}^{q}(\frac{S_{a}^{2}}{\sigma_{j}^{2}})+\lambda \end{array}$$ (5a)

$$\begin{array}{l} f(v|ELSE)\propto f(\sigma_{j}^{2}|S_{a}^{2},v)f(v)\\ =\prod_{j=1}^{q}\frac{{\left(vS_{a}^{2}/2\right)}^{v/2}\exp(-\frac{vS_{a}^{2}}{2\sigma_{j}^{2}})}{\Gamma (v/2)\;*\;{\left(\sigma_{j}^{2}\right)}^{1+v/2}}\;*\;v^{3}\exp(-v)\\ \propto v^{3}{\left(v/2\right)}^{qv/2}{\left[\Gamma (v/2)\right]}^{-q}\exp(-\eta v)\\ \eta =\frac{1}{2}\sum_{j=1}^{q}\ln(\frac{\sigma_{j}^{2}}{S_{a}^{2}})+\frac{1}{2}\sum_{j=1}^{q}(\frac{S_{a}^{2}}{\sigma_{j}^{2}})+1 \end{array}$$ (5b)

In BayesFA, *q* was equal to the total number of SNPs (*P*), while in BayesFCπ and BayesFB, *q* denotes the number of non-zero SNP effects in the current iteration. *λ* was set to 0.25. The posterior distribution of *v* in BayesFB (5b) was similar to BayesFCπ and BayesFA.

To sample *v* in our modified methods, we applied efficient accept-reject [19,20] sampling method. We supposed that *f*(*x*) was a target distribution function as a substitute for *f*(*v*|*ELSE*)(5a). A value *c* was sampled from a standard distribution function *g*(*x*). The appropriate value for *v* was determined by the probability of acceptance *f*(*c*)/M*g(*c*). M was the maximum value of a function *f*(*x*)/*g*(*x*). Thus, the crucial step for sampling *v* was to obtain the maximum value M.

According to the accept-reject algorithm, the target distribution was as follows,

$$f(x;q,\eta )={\left(x/2\right)}^{qx/2}{\left[\Gamma (x/2)\right]}^{-q}\exp(-\eta x)$$ (6)

$$\eta =\frac{1}{2}\sum_{j=1}^{q}\ln(\frac{\sigma_{j}^{2}}{S_{a}^{2}})+\frac{1}{2}\sum_{j=1}^{q}(\frac{S_{a}^{2}}{\sigma_{j}^{2}})+\lambda \geq \frac{1}{2}q+\lambda$$ (7)

And proposal distribution was

g(x;θ)=θexp(−θx) (8)

Here *θ* > 0 is the parameter of the exponential distribution. A probability density function was constructed by taking the logarithm of *f*(*x*)/*g*(*x*).

$$\begin{array}{l} Q(x,\theta ;q,\eta )=\ln[f(x;q,\eta )]/g(x;\theta )\\ =(qx/2)\ln(x/2)-q\ln[\Gamma (x/2)]+(\theta -\eta )x-\ln(\theta ) \end{array}$$ (9)

The first derivative of *x* and *θ* was

∂Q(x,θ;q,η)/∂x=(q/2)[ln(x/2)+1−ψ(x/2)]+(θ−η) (10)

∂Q(x,θ;q,η)/∂θ=x−1/θ (11)

Where *ψ*(*x*) was a Digamma function and *ψ*(*x*) = *d*ln[Γ(*x*)]/*dx*.

The maximum M was calculated using Eqs (10) and (11) equal to zero. With *θ* = 1/*x* obtained from the Eq (11), we defined *h*(*x*) using the substitution of *θ* in Eq (10).

h(x)=∂Q(x,θ;q,η)/∂x=(q/2)[ln(x/2)+1−ψ(x/2)]+1/x−η (12)

Given that ln(*x* / 2) + 1 − *ψ*(*x* / 2) was a decreasing function, we could find *h*(*x*) was a decreasing function. The range of function (12) was provided below.

$$\underset{x\to 0}{\lim}h(x)=+\infty$$ (13)

$$\underset{x\to +\infty }{\lim}h(x)=q/2-\eta$$ (14)

In Eq (7), $\eta \geq \frac{1}{2}q+\lambda$, when *x* tended to positive infinity, *h*(*x*) ≤ 0 was obtained through Eq (14). A value that made *h*(*x*) equal to zero with the range less than zero to positive infinity should be existed. This value was assumed to be x^*^ that was used to obtain the maximum value M, hence the function *Q*(*x*,*θ*;*q*,*η*) obtained the maximum value M with *x* being equal to x^*^, and *θ* was equal to 1 / x^*^. To calculate x^*^, we used the algorithm of exponential dichotomy [21].

Full conditional posterior distribution of $S_{a}^{2}$ in BayesFA, BayesFB and BayesFCπ was built in the same way as for BayesDπ. BayesFCπ used the same full conditional posterior distribution of π (16) as in BayesCπ.

$$\begin{array}{l} f(S_{a}^{2}|ELSE)\propto {\left(S_{a}^{2}\right)}^{qv/2}\exp(-\frac{vS_{a}^{2}}{2}\sum_{j=1}^{q}\frac{1}{\sigma_{j}^{2}})\exp(-S_{a}^{2})\\ \left(S_{a}^{2}|ELSE)∼Gamma(\frac{1}{2}vq+1,1/(\frac{v}{2}\sum_{j=1}^{q}\frac{1}{\sigma_{j}^{2}}+1)\right) \end{array}$$ (15)

(π|ELSE)∼Beta(P−q+1,q+1) (16)

### Simulation study

Simulation population structure: All starting with a base population of 100 animals (50 males and 50 females), 2000 non-overlapping historical generations with the same population size denoted as generation -1999 to generation 0 were simulated. In the base population and each historical generation, 50 males randomly mated with 50 females, and each mating produced two offspring (1 male and 1 female). Six additional generations were simulated after the 2000 historical generations, which formed generation 1 to generation 6. The population size was expanded from 100 to 2000 in generation 1. The process involved 50 males and 50 females from generation 0 randomly mating, and 40 progenies (20 males and 20 females) were produced by each mating pair. Then 50 males were randomly selected from the 500 males as the sires of the next generation, and different population sizes of females (250, 500, and 1000) were used as dams in generations 1–5. Finally, three population sizes (500, 1000 and 2000) from generations 2 to 6 were obtained by each male randomly mating 10 females, and each female produced two offspring. The simulation program was same to that of previous study [13].

Genomic structure: The frequently used mutation-drift equilibrium (MDE) model was applied to simulate whole-genome data. Five chromosomes were simulated, and each contained 1 Morgan with 2000 SNPs that were randomly located in the genome. There was a potential quantitative trait locus (QTL) between two SNPs, so 10000 SNPs produced 9995 potential QTL in total. The true QTL effects, just as the allele substitution effects, were drawn from standard normal distribution as typically done [14,22]. To evaluate the predictive accuracies of modified methods, we set different scenarios by the number of QTL (5, 50, 200 and 500) and heritability (0.1, 0.3, 0.5, and 0.8).

In simulation, genotypes and true breeding values (TBVs) were assigned to each animal from generation 1 to 6. Phenotypic records were assigned only to animals in generation 1 (experimental group), and the animals in generation 2–6 were validation groups. Finally, forty-eight combinations were obtained by different genetic architecture considering reference population size, number of QTL and heritability. Among them, 10 replicates were simulated for each scenario.

### Real dataset

The resource population was established in Ulgai, Xilingole League, Inner Mongolia of China. All the young Simmental beef cattle were born between 2008 and 2013. After weaning, Simmental cattle were moved to Beijing Jinweifuren cattle farm for fattening and put under the same feeding and management conditions. Each animal was measured for growth and developmental traits on time until its slaughter when they were 18 to 24 months of age. Live weight was measured after 24 hours of fasting. During the period of slaughter, carcass traits and meat quality traits were measured according to the Institutional Meat Purchase Specification for fresh beef guidelines. Three response variables (carcass weight, live weight and tenderloin weight) were analyzed in this study. Systematic environment factors including farm, year of measurement and age at slaughter (seasons) effects were adjusted in the mixed linear model. Genetic parameters were calculated using residual maximum likelihood method in animal model using G matrix [23].

In total, 1173 Simmental cattle were genotyped with Illumina Bovine 770K SNP BeadChip. Before statistical analysis, SNP quality control was pre-processed as following: PLINK v1.07 [24] software was used to select SNPs based on minor allele frequency (>0.05), proportion of missing genotypes (<0.05), Hardy-Weinberg equilibrium (p>10^−6^). After quality control, any missing genotypes were replaced with the average value (on a 0–2 scale) for each SNP, and the final data consisted of 1136 Simmental cattle and 697,769 SNPs in the autosomes.

### Cross-validation procedure

To assess the predictive accuracies of modified methods in our study, we explored cross-validation methods using real data. Overall, 1136 Simmental cattle were divided into a reference and validation population. Phenotypes of the animals in the validation set were masked to be unknown. Two cross-validation methods were used to select the reference population [25]. The first method was fivefold cross-validation (random masking), in which genotyped cattle were first random divided into five groups. The whole procedure was repeated 10 times. Each time, the data excluded one group to reference on the remaining four groups to estimated marker effects, which were used to predict GEBVs of individuals from the validation population. Therefore, four-fifth populations (n = 909) were randomly sampled as the reference set, and the remaining one-fifth (n = 227) of the population as the validation set. This resulted in every cattle having predicted GEBVs obtained without using its own information. The second method was to select animals based on their years of birth (cohort masking). Animals that were born before 2012 were taken as the reference population (n = 824) and the animals born in 2012 and 2013 as the validation set (n = 312). To evaluate the accuracy of prediction, we estimated the correlation between GEBVs and true genetic values in validation subset following previous study [26].

### Estimation of SNP effects and hyperparameters

We treated two hyperparameters as unknown and estimated them in both simulation and real data. Markov chains were run for 50,000 cycles of iterations. The first 10,000 were discarded as burn-in. In BayesB and BayesFB, 100 additional cycles of Metropolis Hastings sampling were performed for the variances of marker effects. In the modified methods, every MCMC cycle consisted of Gibbs sampling and accept-reject sampling steps. Scale parameter was sampled by the Gibbs sampling process, and degrees of freedom were acquired by efficient accept-reject scheme. A stable estimation was obtained in these sufficient iterations and little improvement could be made in higher settings. All the samples of SNP effects and the two variable hyperparameters were averaged after burn-in to obtain the estimates. In simulation, π was defined as the number of QTL divided by the total number of loci in BayesB, BayesFB, BayesCπ and BayesFCπ. In the real analysis, π was set as 0.996 for BayesB and BayesFB, and 0.8 for BayesCπ and BayesFCπ.

### Calculation of GEBVs

GEBVs for animals were calculated as the sum of all SNP effects, according to their marker genotypes. The GEBV of animals was calculated as

$$gEBV_{i}=\sum Z_{ij}\alpha_{j}$$ (17)

*Z*_*ij*_ was a genotype for SNP *j* of animal *i*, and *α*_*j*_ was the posterior mean of the *j*-th SNP effect.

## Results

### Simulation study

#### Estimation of SNP effects

**Fig 1:**
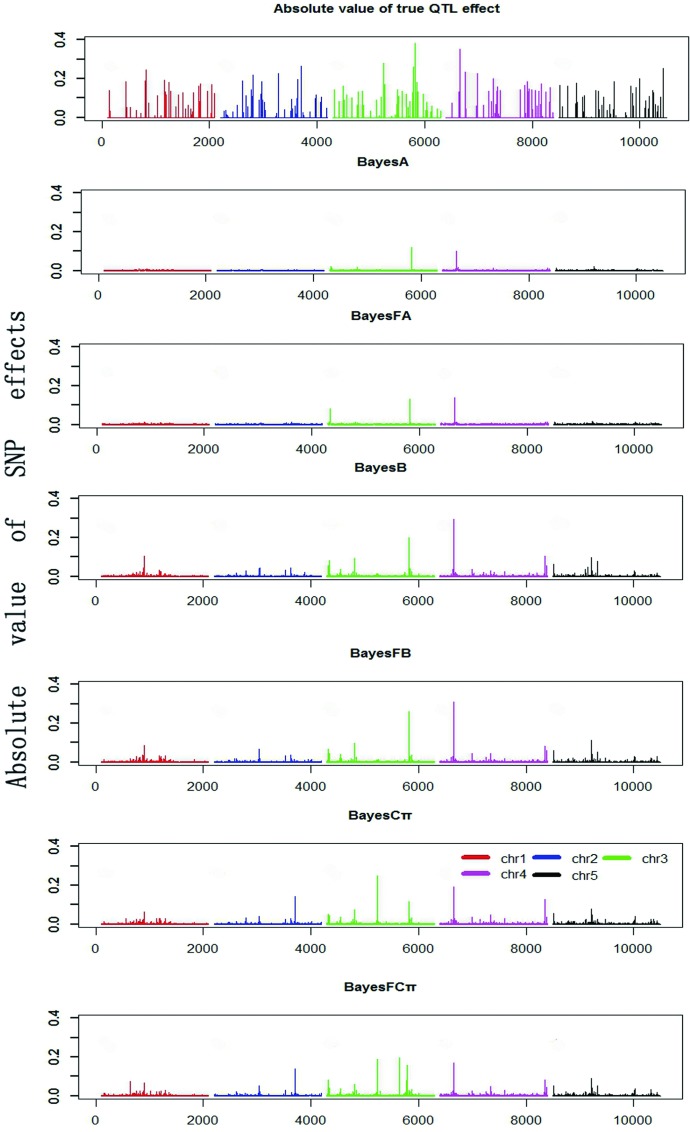
Absolute value of simulated true QTL effects and SNP effects estimated using six Bayesian models from a randomly selected replication (population size = 1000, number of QTL = 200, *h*^*2*^ = 0.5). The absolute values of the SNP effects were estimated using BayesA, BayesFA, BayesB, BayesFB, BayesCπ, and BayesFCπ, respectively.

To compare the difference of SNP effects estimated by the six Bayesian models, we calculated the absolute SNP effects (Fig 1). We found the absolute simulated QTL effects ranged from 0 to 0.38, when reference population size was 1000, the number of QTL was 200 and heritability was 0.5. While the absolute estimated SNP effects varied from 0 to 0.12 for BayesA, 0–0.14 for BayesFA, 0–0.30 for BayesB, 0–0.31 for BayesFB, 0–0.25 for BayesCπ, and 0–0.20 for BayesFCπ. BayesFB acquired highest absolute SNP effects value than other methods.

#### Accuracies of GEBVs in generation 1–6

To estimate the predictive accuracies of the modified methods comparing with their counterparts, we analyzed the simulation datasets with different genetic architectures. Generally, the predictive accuracies of BayesFA and BayesFCπ performed better than their counterparts in all generations. However, the predictive accuracies between BayesFB and BayesB were very similar. In generation 1 with population size of 1000, different number of QTL and heritability, the mean accuracies of GEBVs estimated by GBLUP generated the lowest accuracy (Table 1). In contrast, BayesFA and BayesFCπ improved the predictive accuracy by 0.2–2.5% and 0.2–1.5%, respectively, when compared with their counterparts. Meanwhile, in generation 2–6 with population size of 1000, QTL of 200, and heritability of 0.5, BayesFA and BayesFCπ improved the predictive accuracies by 0.6–1.7% and 0.4–1.1%, respectively (Table 2).

**Table 1:** Predictive accuracies of GEBVs estimated using GBLUP and six Bayesian methods in generation 1 with a population size = 1000, different number of QTL and heritability.

	QTL = 5				QTL = 50				QTL = 200				QTL = 500			
*h*^*2*^	0.1	0.3	0.5	0.8	0.1	0.3	0.5	0.8	0.1	0.3	0.5	0.8	0.1	0.3	0.5	0.8
GBLUP	0.715	0.834	0.888	0.95	0.626	0.798	0.87	0.945	0.568	0.772	0.858	0.942	0.526	0.762	0.854	0.941
BayesA	0.780	0.879	0.921	0.967	0.621	0.789	0.868	0.948	0.566	0.777	0.863	0.942	0.532	0.768	0.856	0.94
BayesFA	0.805	0.902	0.937	0.983	0.637	0.807	0.877	0.954	0.579	0.790	0.871	0.949	0.549	0.773	0.858	0.943
BayesB	0.766	0.894	0.923	0.975	0.631	0.793	0.883	0.948	0.565	0.790	0.865	0.944	0.554	0.766	0.855	0.939
BayesFB	0.768	0.898	0.928	0.978	0.636	0.801	0.884	0.951	0.568	0.794	0.87	0.951	0.563	0.768	0.857	0.945
BayesCπ	0.799	0.886	0.924	0.965	0.631	0.799	0.882	0.954	0.564	0.792	0.867	0.946	0.549	0.769	0.856	0.94
BayesFCπ	0.814	0.896	0.931	0.973	0.64	0.809	0.883	0.957	0.569	0.794	0.869	0.95	0.561	0.774	0.858	0.942

Values are means of predictive accuracy from 10 replicates. GBLUP, genomic best linear unbiased prediction; GEBV, genomic estimated breeding value; QTL, quantitative trait loci

**Table 2:** Predictive accuracies of GEBVs estimated using GBLUP and six Bayesian methods in generations 2–6 with a population size = 1000, QTL = 200, *h*^*2*^ = 0.5.

Methods	Generation 2	Generation 3	Generation 4	Generation 5	Generation 6
GBLUP	0.745±0.008	0.711±0.011	0.680±0.019	0.633±0.028	0.602±0.021
BayesA	0.754±0.013	0.719±0.021	0.689±0.015	0.640±0.022	0.605±0.011
BayesFA	0.766±0.015	0.736±0.111	0.695±0.013	0.649±0.017	0.611±0.012
BayesB	0.759±0.018	0.720±0.015	0.693±0.011	0.647±0.011	0.606±0.020
BayesFB	0.767±0.012	0.725±0.013	0.696±0.015	0.651±0.008	0.610±0.014
BayesCπ	0.757±0.008	0.720±0.014	0.693±0.012	0. 649±0.011	0.606±0.012
BayesFCπ	0.766±0.013	0.731±0.017	0.695±0.014	0.653±0.024	0.610±0.017

Values are means and standard deviations of predictive accuracy from 10 replicates.

#### Effect of heritability and QTL on prediction

To evaluate the effects of heritability and QTL on predictive accuracy, we simulated four different heritabilities (0.1, 0.3, 0.5 and 0.8) and numbers of QTL (5, 50, 200 and 500). In Table 1, when the genetic architecture of QTL was the same, we observed the predictive accuracies dropped with a decrease in heritability. On the other hand, the predictive accuracies increased along with decreasing the number of QTLs under the same heritability. Furthermore, for a small number of QTL (QTL = 5) and low-heritability (*h*^*2*^ = 0.1), the predictive accuracies improved with the highest (2.5% and 1.5%) for BayesFA and BayesFCπ compared to their counterparts, respectively. Additionally, the predictive accuracies for high (*h*^*2*^ = 0.8) and medium (*h*^*2*^ = 0.5) heritability were not robust than those for low heritability.

#### Inference on key hyperparameters

**Fig 2:**
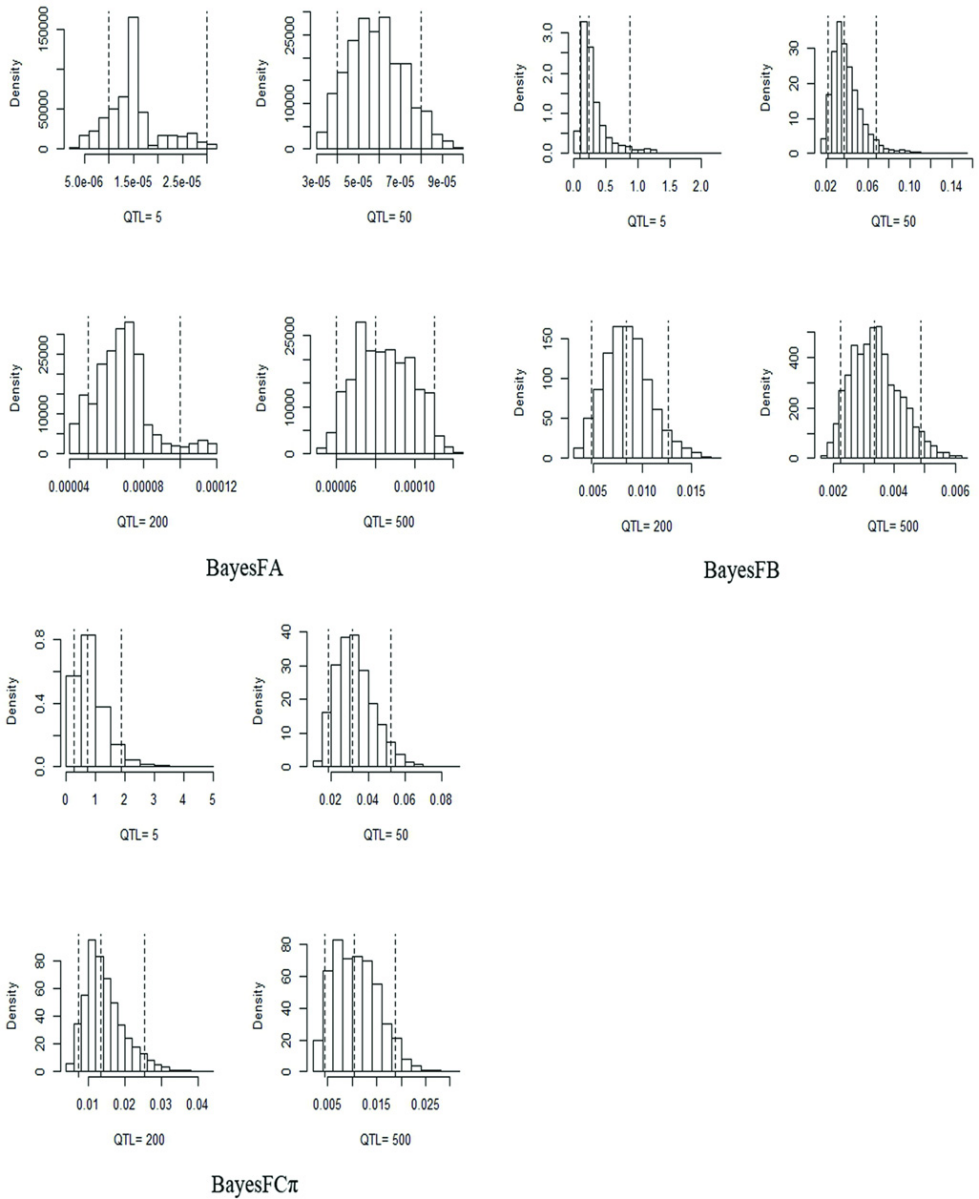
Histogram of the posterior samples for the scale parameters of the Inv-χ^2^ prior on variances with a population size = 1000, QTL = 5, 50, 200, 500 and *h*^*2*^ = 0.5 in BayesFA, BayesFB and Bayes FCπ, respectively. The dotted lines represent the posterior 5, 50 and 95% quantiles.

**Fig 3:**
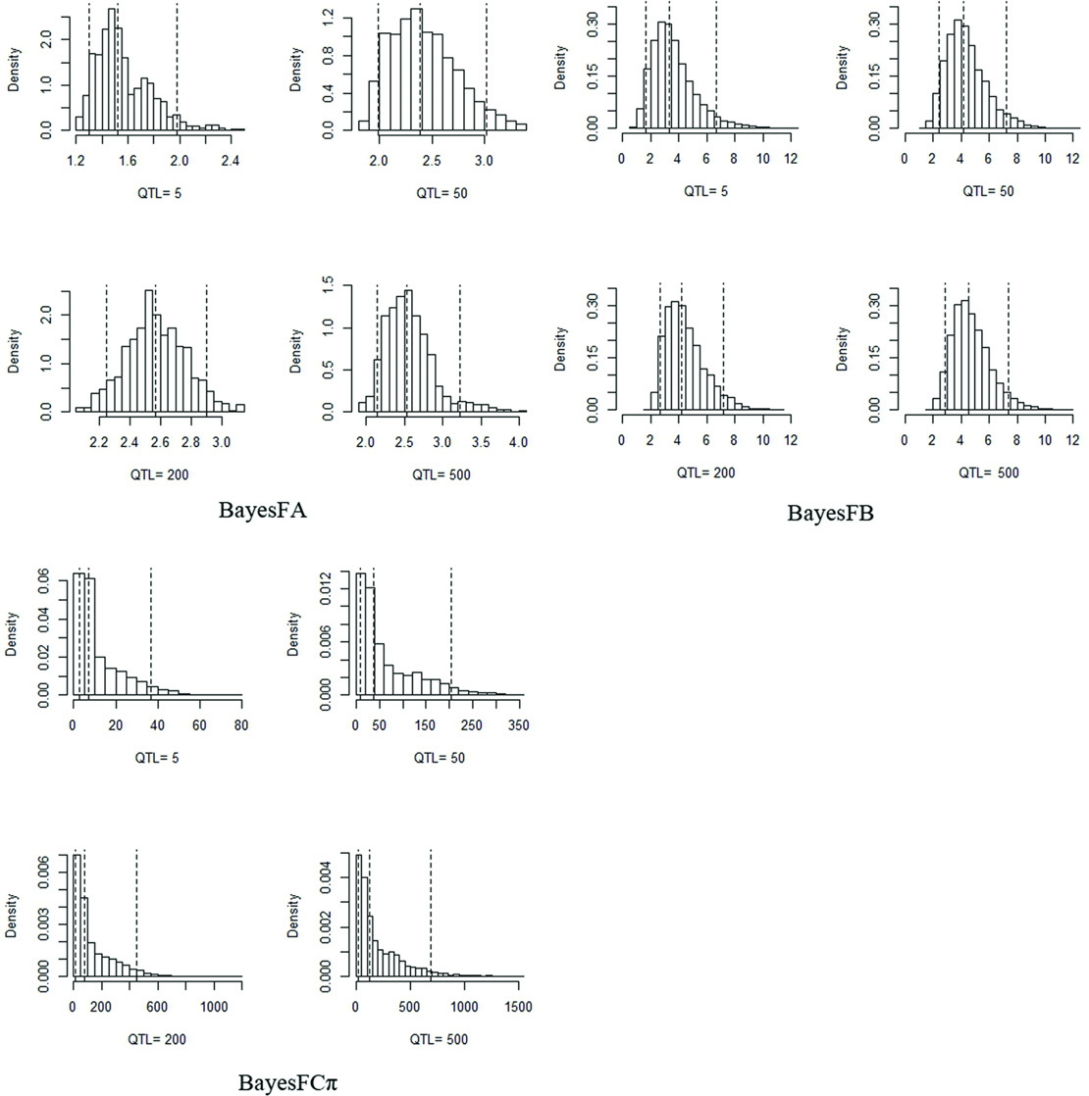
Histogram of the posterior samples for the degrees of freedom of the Inv-χ^2^ prior on variances with a population size = 1000, QTL = 5, 50, 200, 500 and *h*^*2*^ = 0.5 in BayesFA, BayesFB and BayesFCπ, respectively. The dotted lines represent the posterior 5, 50 and 95% quantiles.

For simulated data, QTL effects were drawn from the standard normal distribution. These were produced by the process of recombination over thousands of generations in terms of the relationships between the number of QTL and *v* or $S_{a}^{2}$. It was difficult to surmise the “true” value of key hyperparameters. However, we found that estimates of $S_{a}^{2}$ were inversely related to the number of QTL for BayesFB and BayesFCπ (Fig 2). Under population size of 1000, QTL of 200 and heritability of 0.5, the estimates of $S_{a}^{2}$ for the three modified methods were different, with posterior median at 7e-5, 0.008, 0.01 and the 95% posterior interval of [5e-5, 1e-4], [0.0048, 0.012], [0.0073, 0.025] in BayesFA, BayesFB, BayesFCπ, respectively. Histograms of the posterior samples for the degrees of freedom obtained by the modified methods with population size of 1000 and heritability of 0.5 were shown in Fig 3. These values were markedly different when QTL = 200, with the posterior median at 2.6, 4.3, 118.54 and 95% posterior interval of [2.25, 2.9], [2.6, 7.2], [14.9, 451.9] in BayesFA, BayesFB, BayesFCπ, respectively. For the three modified methods, it was clear that the posterior median of *v* increased with the number of QTLs.

### Real dataset

Heritability estimates of three traits were 0.38, 0.43 and 0.47 for CW, LW and TW, respectively (Table 3). To assess the predictive accuracies, we applied two different cross-validation methods (random masking and cohort masking) in Simmental population. The predictive accuracies of GBLUP and Bayesian methods using the random masking method were superior to those in the cohort masking method. BayesFA showed slightly higher predictive accuracy than BayesA in the two cross-validation methods, and the accuracy improved by 0.4% to 1.2%. The predictive accuracies of BayesFB outperformed those of BayesB by 0.3% to 1%. Meanwhile, the predictive ability of BayesFCπ obviously outperformed BayesCπ with the highest increase (3.8%) of LW in cohort masking. Average posterior means of the hyperparameters estimated by modified methods across the 10 replicates were shown in Table 4. These hyperparameters were obviously different to their previous ones.

**Table 3:** Heritability estimation and accuracies of GEBVs estimated using GBLUP and six Bayesian methods for three traits.

Trait	*h*^*2*^	CV	GBLUP	BayesA	BayesFA	BayesB	BayesFB	BayesCπ	BayesFCπ
CW	0.38	RM	0.470	0.486	0.498	0.487	0.491	0.483	0.488
		CM	0.413	0.413	0.422	0.411	0.414	0.402	0.409
LW	0.43	RM	0.526	0.576	0.582	0.594	0.602	0.598	0.596
		CM	0.468	0.474	0.478	0.460	0.470	0.412	0.450
TW	0.47	RM	0.540	0.560	0.570	0.566	0.569	0.561	0.569
		CM	0.474	0.467	0.474	0.478	0.485	0.475	0.483

RM, Random masking is the first way to mask phenotypes using fivefold cross-validation, and CM, Cohort masking is the second way to mask phenotypes by their birth year. Values are the means of 10 repeats for RM method. Accuracy is evaluated using ${\overset{⌢}{\rho }}_{g\overset{⌢}{g}}=\frac{{\overset{⌢}{\sigma }}_{EBV,GEBV}}{\sqrt{h^{2}\sigma_{p}^{2}\sigma_{GEBV}^{2}}}$. *EBV*, estimated breeding values. $\sigma_{p}^{2}$ is the phenotypic variance estimated from the primary analysis using all genotyped animals in the reference population. *GEBV*, genomic estimated breeding value; GBLUP, genomic best linear unbiased prediction; CW, carcass weight; LW, live weight; TW, tenderloin weight.

**Table 4:** Average posterior means of the hyperparameters (*v*, $S_{a}^{2}$) based on BayesFA, BayesFB and Bayes FCπ analyses for three traits.

Methods	Hyperparameters	CW	LW	TW
BayesFA	*v*	4.08(0.21)	4.88(0.21)	5.17(0.13)
	$S_{a}^{2}$	1.66 e-3 (1.4 e-4)	6.87 e-4 (2.6 e-5)	6.52e-6(8.12e-7)
BayesFB	*v*	4.92(0.28)	4.04(0.16)	4.80(0.17)
	$S_{a}^{2}$	0.869(0.026)	0.372(0.016)	9.02e-5(8.12e-6)
Bayes FCπ	*v*	11.35(1.02)	8.43(0.85)	12.53(1.06)
	$S_{a}^{2}$	0.179(0.014)	1.762(0.105)	0.081(0.003)

Empirical standard deviations across 10 replicates are provided in parentheses.

## Discussion

Genomic selection has revolutionized cattle breeding by greatly increasing the predictive accuracies of genetic merit for young animals and shortening the generation interval. In our study, we performed genomic prediction in Chinese Simmental beef cattle with addressing the impact of *v* and $S_{a}^{2}$ on predictive accuracies. Gianola proposed that both the degrees of freedom and the scale parameters can affect the shrinkage of SNP effects [27]. To address these issues, they applied a full hierarchical approach to estimate the optimal priors instead of assigning fixed values [16,17]. Based on treating $S_{a}^{2}$ as unknown with Gamma (1, 1) in BayesDπ [7], we further treated degree of freedom as unknown and gave an inappropriate prior distribution in BayesFA, BayesFB and BayesFCπ. To illustrate the impact of these two hyperparameters on genomic predictive accuracy, we simulated different scenarios including population sizes, numbers of QTL and heritability. Finally, cross-validation analysis using 1136 Simmental beef cattle showed that predictive ability of BayesFCπ clearly outperformed BayesCπ with the highest increase (3.8%) for live weight in the cohort masking cross-validation.

For simulation study, Yang *et al*. used antedependence model for whole genomic prediction to explore the relationship between $S_{a}^{2}$ and linkage disequilibrium levels by simulating six different SNP marker densities [17], and found the antedependence models had significantly higher accuracies than their corresponding counterparts at higher LD level with differences exceeding 3%. Habier *et al*. discussed the impact of $S_{a}^{2}$ in BayesDπ, and found it decreased with the increasing number of SNPs in their analysis [7]. In our simulation, when the population size was 1000 and *h*^*2*^ = 0.5, the value decreased with the increasing number of QTL in BayesFB and BayesFCπ as shown in Fig 2. For degrees of freedom, Jia *et al*. developed a full hierarchical approach to estimate *v* [28] and found optimal marker-effect variance depended on the genetic architecture of the trait. We assumed inappropriate priors for *v* in BayesFA and BayesFCπ with exponential distribution, and BayesFB with gamma distribution. The larger degrees of freedom sampled by efficient accept-reject can result in greater impact on the posterior SNP variances inference. The posterior median degrees of freedom estimated by BayesFA, BayesFB and BayesFCπ were 2.6, 4.3, 118.54, respectively, but different from the fixed value 4.2 as suggested by Meuwissen *et al*. [14]. Therefore, a large value for the degrees of freedom may not indicate better prediction. In this study, BayeaFA estimated smaller degrees of freedom (2.6) than in BayesA (4.2), while BayesFA show higher predictive accuracy than in BayesA.

For real dataset, Simmental beef cattle was partitioned into training and validation data sets, cross-validation analyses were carried out using CW, LW and TW traits to assess the accuracies of genomic prediction. Previous studies have conducted genomic selection of CW and LW traits in other Simmental cattle [29–31]. We found the estimated heritability of CW (0.38) in our study was slightly lower than Mahdi Saatchi’s study (0.4) [30]. In Table 3, BayesFA and BayesFB showed higher accuracy of GEBVs than their counterparts for all traits using the two cross-validation methods. BayesFCπ increased the accuracy as compared with BayesCπ in three traits except for LV in random masking method. The degrees of freedom and scale parameters in the modified methods were treated in inappropriate priors to be estimated from the data, which have significant impacts of locus-specific variances elaborated by Habier *et al*. [7]. Therefore, the predictive accuracies of these modified methods were superior to their conventional counterparts. Using high density SNP chip, we found BayesA performs markedly better than other conventional methods (BayesB and BayesCπ) for LW in cohort masking cross-validation method despite the statistical drawback of BayesA as described by Gianola *et al*. [8].

The proposal distribution of locus-specific variance is an inverse chi-square distribution with parameters *v* and $S_{a}^{2}$. In each iteration, the modified methods sampled new degrees of freedom and scale parameters. These variable hyperparameters could influence the sampling of variances of SNP effects, which may induce higher predictive accuracies than their conventional counterparts. Two hyperparameters estimated in our study were different from Yang *et al*. [17]. Their estimates of the degrees of freedom were extremely large (>15) for anteBayesA and anteBayesB methods with heterogeneous stock mice data. These differences may be caused by different prior assumption, sampling algorithm and dataset. In contrast, our study showed larger values for the degrees of freedom only in BayesFCπ.

Due to the additional sampling the variable *v* and $S_{a}^{2}$ in each iteration, the computing time in our methods was longer than their counterparts, respectively, *i*.*e*., with population size of 1000, QTL = 200, *h*^*2*^ = 0.5 using 3.3GHz, *Intel core(TM) i3-2120* processor, computing time for 50000 iterations was 2.39, 2.56, 5.08, 5.36, 2.12 and 2.29 hr for BayesA, BayesFA, BayesB, BayesFB, BayesCπ and BayesFCπ, respectively. However, these modified methods produced higher predictive accuracies than their conventional counterparts. For the posterior distribution in a complicated non-standard form, the degrees of freedom sampled by the efficient accept-reject algorithm may produce anticipated results.

In conclusion, we performed genomic prediction for live weight, carcass weight and tenderloin weight in Chinese Simmental beef cattle. Our study indicated the degrees of freedom and scale parameters could slightly impact the predictive accuracies in both simulation and real dataset.
